# Performance of the APOP-screener for predicting in-hospital mortality in older COVID-19 patients: a retrospective study

**DOI:** 10.1186/s12877-022-03274-2

**Published:** 2022-07-15

**Authors:** Marleen G. A. M. van der Velde, Merel J. van der Aa, Merel H. C. van Daal, Marjolein N. T. Kremers, Carolina J. P. W. Keijsers, Sander M. J. van Kuijk, Harm R. Haak

**Affiliations:** 1 Department of Internal Medicine, Máxima MC, De Run 4600, Veldhoven-Eindhoven, 5504 DB The Netherlands; 2 Department of Health Services Research, and CAPHRI School for Public Health and Primary Care, Aging and Long Term care Maastricht, Maastricht, the Netherlands; 3 Department of Geriatric Medicine, Jeroen Bosch Hospital, ‘s Hertogenbosch, the Netherlands; 4 Department of Emergency Medicine, Sint Jans Gasthuis, Weert, The Netherlands; 5 Department of Clinical Epidemiology and Medical Technology Assessment, Maastricht University Medical Center, Maastricht, The Netherlands; 6 Department of Internal Medicine, Division of General Internal Medicine, Maastricht University Medical Center, Maastricht, the Netherlands

**Keywords:** COVID-19, SARS-Cov2, Frailty, Predictive, Mortality

## Abstract

**Background:**

A variety of prediction models concerning COVID-19 have been proposed since onset of the pandemic, but to this date no gold standard exists. Mortality rates show a sharp increase with advancing age but with the large heterogeneity of this population in terms of comorbidities, vulnerability and disabilities, identifying risk factors is difficult. Therefore, we aimed to research the multidimensional concept of frailty, measured by the Acute Presenting Older Patient (APOP)-screener, as a risk factor for in-hospital mortality in older COVID-19 patients.

**Methods:**

All consecutive patients of 70 years or older, with a PCR confirmed COVID-19 infection and a completed APOP-score, presenting at the Emergency Department (ED) of the Jeroen Bosch Hospital, the Netherlands, between February 27th 2020 and February 1st 2021 were retrospectively included. We gathered baseline characteristics and scored the CCI and CFS from patient records. The primary outcome was in-hospital mortality.

**Results:**

A total of 292 patients met the inclusion criteria. Approximately half of the patients were considered frail by the APOP or CFS. 127 patients (43.5%) scored frail on the CFS, 158 (54.1%) scored high risk on the APOP-screener. 79 patients (27.1%) died during their hospital admission. The APOP-screener showed a significantly elevated risk of in-hospital mortality when patients scored both high risk of functional and evidence of cognitive impairment (OR 2.24, 95% 1.18–4.25). Significant elevation of in-hospital mortality was found for the high CCI-scores (≥ 5)(OR 1.78, 95% 1.02–3.11), but not for the highest CFS category (5–9, frail) (OR 1.35, 95% 0.75–2.47). The discriminatory performance of the APOP, CFS and CCI were comparable (AUC resp. 0.59 (0.52–0.66), 0.54 (0.46–0.62) and 0.58 (0.51–0.65)).

**Conclusion:**

Although the elevated risk for in-hospital mortality found for the most frail patients as scored by the APOP, this instrument has poor discriminatory value. Additionally, the CFS did not show significance in predicting in-hospital mortality and had a poor discriminatory value as well. Therefore, treatment decisions based on frailty or comorbidities alone should be made with caution. Approaching the heterogeneity of the older population by adding frailty as assessed by the APOP-score to existing prediction models may enhance the predictive value of these models.

**Supplementary Information:**

The online version contains supplementary material available at 10.1186/s12877-022-03274-2.

## Key points

- A variety of prediction models concerning COVID-19 have been proposed since onset of the pandemic, but to this date no gold standard exist.
- We aimed to evaluate the multidimensional concept of frailty, measured by the Acute Presenting Older Patient (APOP)-screener, as a risk factor for in-hospital mortality in older COVID-19 patients. We compared this to frailty as scored by the Clinical Frailty Scale (CFS) and the Charlson Comorbidity Index (CCI).
- Although the elevated risk for in-hospital mortality found for the most-frail patients as scored by the APOP, and the most comorbid patients as scored by the CCI, they both had poor discriminatory value.
- For predicting in-hospital mortality, the CFS, as divided into three categories, did not show prognostic value.
- Treatment decisions based on frailty or comorbidities alone should be made with caution.
- Approaching the heterogeneity of the older population by adding frailty as assessed by the APOP-score to existing prediction models may enhance the predictive value of these models.

## Background

The emergence of severe acute respiratory syndrome coronavirus 2 (SARS-CoV-2) has put an enormous burden on health care systems throughout the world. The treatment of the acute respiratory distress syndrome caused by COVID-19, consists of aggressive supportive care such as mechanical ventilation. When deciding to administer these treatments, it could be of value to identify patients for whom the burden of the treatment outweigh the benefits. In addition, when in times of shortage of care or with the burden of catching up on postponed regular care, predicting which patients are at risk of poor outcomes after infection can be of great value as it can assist in selecting patients to be allocated to these resources. Since the onset of this pandemic, a lot of research has been published on risk factors of poor outcomes, such as Intensive Care Unit (ICU)-admittance and mortality. Major risk factors include age, male sex, hypertension, smoking, diabetes mellitus, cardiovascular disease, obesity, as well as different lab abnormalities [1–7]. Various prediction models for mortality have been proposed, using a variety of information such as age, vital signs and specific laboratory results. In the variety of prediction models concerning COVID-19, a division can be made in either newly developed and validated models most often consisting of multiple variables, and evaluation of the prognostic value of pre-existing assessment tools as the Clinical Frailty Scale (CFS) and Charlson Comorbidity index (CCI). To this date, a sufficiently accurate model for predicting poor outcomes in COVID-19 does not exist and because of the difficulties in identifying risk factors, most often restrictive recommendations have been directed broadly to people older than 70 years or older, irrespective of risk-factors, health or activity status [8].

With advancing age comes a higher prevalence of chronic conditions. A widely accepted scoring method for measuring comorbidities is the CCI. The total score of the CCI consists in a simple sum of the weights of 19 different morbidities, with a higher score indicating more severe comorbid conditions and a greater mortality rate [9]. The CCI has been evaluated for predicting mortality in COVID-19 patients and proven independently associated with mortality [8, 10, 11].

Worldwide the older population is characterized by a large heterogeneity in terms of comorbidities, functional and cognitive status. However, irrespective of age and comorbidities some individuals age faster and are more vulnerable or susceptible to disease and disability. To recognize this condition, the concept of frailty has been introduced. Frailty proved to be an important predictor of mortality in older adults in general [12]. In a recently published study, frailty was also independently associated with higher in-hospital mortality in older hospitalised COVID-19 patients [13–15]. There are various instruments available to measure frailty in patients presenting at the ED. [16] The Clinical Frailty Scale (CFS), is such a judgement-based frailty tool that evaluates specific domains as function, cognition and comorbidity to generate a score ranging from 1 (very fit) to 9 (terminally ill) [17]. The CFS has proven to be of good predictive ability for mortality in variable populations, including the COVID-19 pandemic [13, 18–20].

Another way to quantify frailty in patients visiting the Emergency Department (ED), which has already been implemented in EDs throughout the Netherlands, is the Acute Presenting Older Patient (APOP) [21]. This short questionnaire, which can be completed in only 2 min, consists of questions regarding performance status, medical history, gender and age. The APOP-screener identifies older patients at highest risk for functional decline and mortality, and aids in recognition of cognitive impairment and enhances appropriate care in the ED. This frailty tool is not based on judgement in contrary to the CFS, but is based solely on objective answers from the patient or care-taker. Therefore, the APOP-screener could add value to the measurement of frailty. The predictive value in patients with COVID-19, has yet to be examined.

The aim of this study was to determine whether the APOP-screener, could be used as a tool to accurately predict in-hospital mortality in older patients suffering from COVID-19. We hypothesise that a high risk APOP-score will be a significant predictor for mortality in older patients with COVID-19. The secondary aim was to compare the predictive value of the APOP-screener with the Charlson Comorbidity Index and the Clinical Frailty Scale.

## Methods

### Study design and setting

The predictive value of the APOP-screener was studied retrospectively in older ED patients. It was performed in a Dutch teaching hospital, the Jeroen Bosch Hospital (JBZ), with nearly 34.500 patients visiting the ED annually. Given the retrospective design of this study, no informed consent was necessary.

Patient privacy was ensured by pseudonymisation of the data by replacing all identifying variables with a unique study patient code. The medical ethics committee of the Jeroen Bosch Hospital approved this study. The METC number is NW 2020–91.

### Study population

The study sample consisted of consecutive patients, aged 70 years and older, who were presented at the ED, had a complete APOP-screening and were diagnosed with COVID-19 (confirmed PCR nasopharyngeal, sputum or bronchoalveolar lavage examination), between February 27th 2020 and February 1st 2021. There were no exclusion-criteria defined.

### Data collection

Patient selection took place by using CTcue, a self-serviced pseudonymised data-platform, by which the patients files can be searched [22]. Eligible patients were identified by adding specific queries into the search engine, see Supplementary Table 1. These data were retrieved pseudonymised according to our defined inclusion criteria, study parameters and endpoints. We collected baseline characteristics, such as age, gender, living arrangement and comorbidities following the Charlson Comorbidity Index (CCI). Finally, we retrieved the outcome measurements; treatment restrictions and in-hospital mortality. Treatment restrictions were discussed with each patient at the ED by their treating physician and decision was afterwards recorded in the patient file.

### Screening instruments

Frailty was quantified by using the APOP-screener. It is a screening tool that provides an estimation of the risk of functional decline of the patient within 3 months and the presence of cognitive impairment at the time of administration [23, 24]. The APOP-screener consists of 9 short questions within a 2 min time-span. These questions lead to an estimated risk of frailty, expressed in two domains: risk of functional decline and estimated cognitive impairment [23]. The estimated risk is converted to four outcomes: low risk, high risk at functional decline, evidence of cognitive impairment and both risk of functional decline and evidence of cognitive impairment.

Although many frailty assessment instruments are available, relatively few are validated for use in the acute inpatient setting. Before the onset of the pandemic only the APOP-score was used in the ED of the JBZ hospital. When infection rates peaked, concerns about possible need for prioritizing care and ICU-beds were raised. In preparation, the Royal Dutch Medical Association (RDMA) followed worldwide expert opinion and obligated hospitals to asses frailty according to the CFS for every admitted patient. CFS-scores for the included patients were not retrievable at the time of data-collection, thus the CFS score was determined by the researchers based on information provided in the medical records of patients, i.e. functional status, living arrangement and need of care. Frailty status as scored by the CFS, can be accurately assessed by retrospective review of patient records [25–29]. The researchers were trained in assessing CFS and all had appropriate expertise with practical care for older patients. They independently ranked the patients’ frailty according to the CFS scale, and all scores were discussed afterwards until consensus was reached. The CFS ranges from 1 (very fit) to 9 (terminally ill) and is commonly divided into 3 categories (1–3 = fit, 4 = vulnerable, 5–9 = frail) [17, 30, 31].

The CCI was scored by the researchers based on information found in the medical records of the patients. It ranges from 0 until 37, with higher scores indicating more comorbidities [9]. Most studies using CCI for predicting mortality in patients with COVID-19 divide patients using the categories 1–2, 3–4 and > 4 [11, 32]. However, when considering a population with many comorbidities these categories often results in a severely skewed ratio, and are therefore not appropiate [33]. Therefore, the CCI was divided in a low category [1–4] and a high category (≥ 5). For a summary of these instruments, see Table 1.

**Table 1:** Overview of included screening and prediction models

Model	Validated in	Items
APOP [21]	Patients ≥70 years	Acute Presenting Older Patient. Seven predictors that are collected in less than 2 minutes after ED arrival: age, sex, arrival by ambulance, need of regular help, need for help with bathing and showering, hospitalization in the past 6 months, and impaired cognition (defined as having dementia, an incorrect answer on at least one of two 6-CIT questions [“what year is it now?” and/or “say the months in reverse order”], or no data of cognition). Possible result: ‘low risk’, ‘high risk of functional decline’, ‘evidence for impaired cognition’ or ‘high risk of functional decline and evidence for impaired cognition’.
Clinical frailty scale [13, 17–20]	All patients (in critically ill patients; ≥65 years)	Measure of pre-admission health state; 2 weeks prior to admission. Ranges from 1 to 9, with higher values indicating greater frailty: 1 = very fit, 2 = well, 3 = managing well, 4 = very mildly frail (previously ‘apparently vulnerable’), 5 = mildly frail, 6 = moderately frail, 7 = severely frail, 8 = very severely frail and 9 = terminally ill.
Charlson Comorbidity Index [9, 11, 34]	All patients	Measures several comorbidities and combines them with age, resulting in a total score between zero and 37.

*APOP* Acute presenting Older Patient – screening

### Outcomes

The primary outcome was the predictive value of the APOP-screener on in-hospital mortality after ED-presentation. Secondary outcome was the comparison of the predictive value of the APOP-screening compared to the CCI and the CFS.

### Statistical analysis

Baseline characteristics were analysed using descriptive statistics on the observed data. Continuous data are presented as mean and standard deviation (SD) or median and interquartile range (IQR), nominal data are presented as number and percentage. To compare the baseline characteristics between the survivor- and deceased group, *p*-values were calculated by using Pearson’s chi-square or Fisher’s exact test for categorical variables, and the independent-samples t-test or Mann-Whitney U test for continuous variables. To assess the association between in-hospital mortality and the frailty-instruments, odds ratios (ORs) and 95% confidence intervals (CI) were calculated using binary logistic regression, with the determinant ‘low risk’ as reference-category. Subgroup analysis was performed to calculate this association in the no treatment restriction group. To compare the two screenings-instruments, we calculated the area under the receiver operating characteristic curve (AUC) to quantify the discriminatory performance of the screenings models. An AUC of 0.5 corresponds to very poor discriminatory performance, whereas an AUC of 1.0 means perfect discriminative ability. All data were analysed using the software Statistical Package for the Social Sciences (SPSS), version 25 (IBM Corp, New York, USA). Significance was set at 0.05.

## Results

### Baseline characteristics

Table 2 shows baseline characteristics. During the study period, 292 patients were included. The mean age of patients was 79.5 years (SD 6.3), with more male patients (57.5%). The median Charlson Comorbidity Index was 5 (range 1–12), 176 patients had a CCI-score of ≥5 (60.3%). A minority of the patients had a BMI of 30 or above (36, 12.3%), even lower patient numbers suffered from moderate to severe kidney disease (6, 2.1%) as scored by the CCI. (Table 2).

**Table 2:** Patient characteristics at admission to the emergency department

Characteristics	Total *N* = 292	Alive at discharge *N* = 213	In hospital mortality *N* = 79	*p*-value
Days of follow-up (median; IQR)	114 (68–272)	110 (65–271)	118 (73.5–273.5)	0.281
Age (mean ± SD)	79.5 ± 6 3	79.0 ± 6.2	80.7 ± 6.4	0.040
70–74 years	79 (27.1%)	61 (28.6%)	18 (22.8%)	
75–79 years	77 (26.4%)	61 (28.6%)	16 (20.3%)	
80–84 years	68 (23.3%)	47 (22.1%)	21 (26.6%)	
≥ 85 years	68 (23.3%)	44 (20.7%)	24 (30.4%)	
Gender				0.011
Female	124 (42.5%)	100 (46.9%)	24 (30.4%)	
Male	168 (57.5%)	113 (53.1%)	55 (69.6%)	
BMI ≥ 30 (kg/m^2^) Missing 154 (52.7%)	36 (12.3%)	27 (12.7%)	9 (11.4%)	0.836
Moderate to severe kidney failure, in CCI	6 (2.1%)	5 (2.3%)	1 (1.3%)	0.565
Living arrangement before ED Missing 18 (6.1%)				0.182
Home, without homecare	171 (58.6%)	131 (61.5%)	40 (50.6%)	
Home, with homecare	73 (25.0%)	46 (21.6%)	27 (34.2%)	
Home, no further information	14 (4.8%)	11 (5.2%)	3 (3.8%)	
Care/nursing home or other	13 (4.5%)	9 (4.2%)	4 (5.1%)	
APOP^a^				0.071
Low risk on adverse outcomes	134 (45.9%)	107 (50.2%)	27 (34.2%)	
High risk of functional decline	26 (89.0%)	17 (8.0%)	9 (11.4%)	
Evidence of impaired cognition	60 (20.5%)	43 (20.2%)	17 (21.5%)	
High risk of functional decline and evidence of impaired cognition	72 (24.6%)	46 (21.6%)	26 (32.9%)	
Clinical Frailty Scale Missing 18 (6.1%)				0.121
Fit [1–3]	101 (34.6%)	76 (35.7%)	25 (31.6%)	
Vulnerable [4]	46 (15.8%)	38 (17.8%)	8 (10.1%)	
Frail [5–9]	127 (43.5%)	88 (41.3%)	39 (49.4%)	
Charlson Comorbidity Index (median; minimum-maximum) Missing 3 (1.0%)	5 (1–12)	5 (1–12)	5 (3–12)	0.040
CCI 1–4	113 (38.7%)	90 (42.3%)	23 (29.1%)	0.042
CCI ≥ 5	176 (60.3%)	121 (56.8%)	55 (69.6%)	
Treatment restrictions^b^ Missing 30 (10.3%)				
Do not resuscitate	202 (69.2%)	136 (63.8%)	66 (83.5%)	0.020
Do not intubate	195 (66.8%)	129 (60.6%)	66 (83.5%)	0.014
No ICU admission	185 (63.4%)	122 (57.3%)	63 (79.7%)	0.007

*BMI* Body Mass Index, *ED* Emergency Department, *APOP* Acute presenting Older Patient-screening, *GFR* Glomerular filtration rate, *CCI* Charlson Comorbidity Index. Values on some baseline characteristics were missing, numbers and percentages are reported. Unless otherwise specified, data are number of patients (%). Percentages in second column are of total number of patients, the third and fourth column for the specific group (‘alive at discharge’ and ‘in-hospital mortality’

^a^ APOP-score ‘high risk of functional decline and cognitive impairment’, were also scored separately

^b^ Patients could have more than one treatment restriction

**Fig. 1:**
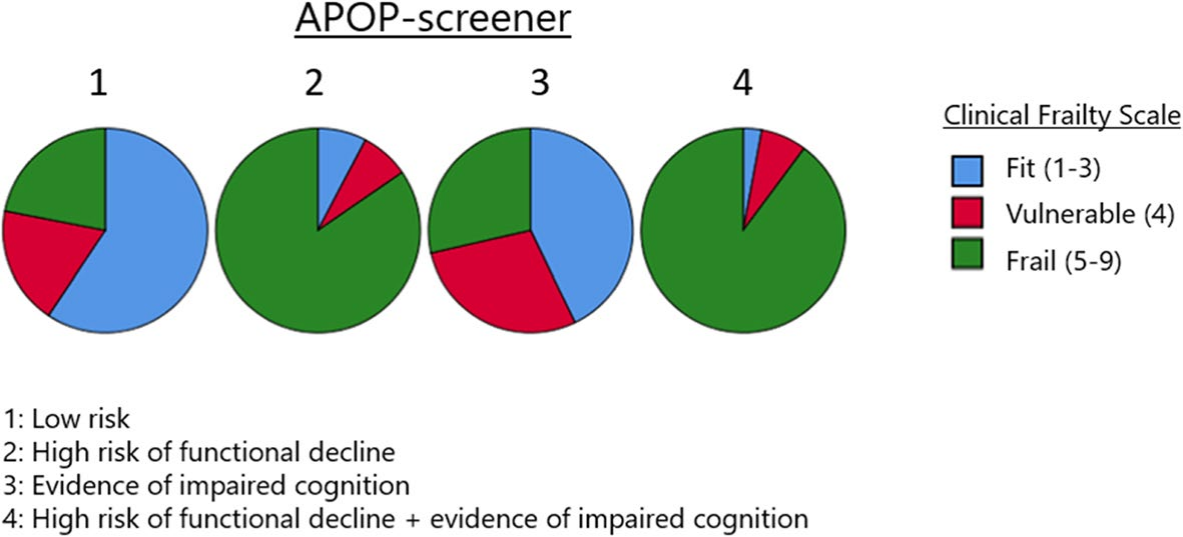
Subdivisions of the clinical frailty scores within the APOP-screener categories. APOP: Acute Presenting Older Patient. The four pie charts correlate with the four possible outcomes of the APOP-screener. The subdivisions within the pie chart consist of the distribution of frailty when assessed following the Clinical Frailty Scale

Approximately half of the patients were considered frail by one or both screening instruments. 127 patients (43,5%) scored frail (scores 5 to 9) on the CFS and 158 (54.1%) scored high risk in the APOP-screener (see Fig. 1). In our population there were no patients with a CFS-score of 9, indicating patients who are terminally ill. The highest and lowest scores of the APOP-screener correlate with corresponding scores of the Clinical Frailty scale. Approximately 75% of the patients with the ‘low risk’ APOP result, score ‘fit’ or ‘vulnerable’ on the CFS. Patients with a high risk of functional decline, on its own or in combination with evidence of cognitive impairment, correspond with higher CFS-scores in our population. When patients score merely high risk on the cognitive domain, the majority falls within the lower categories of the CFS. (see Fig. 1).

In this study population 79 patients (27.1%) died during their hospital stay. These patients were older and more often male, respectively 69.6% vs 53.6%, *p*-value 0.011. In our cohort, a BMI of 30 or above or severe kidney disease did not significantly differ between the patients that deceased during or survived their hospital stay. Overall, most of the patients in the study sample had treatment restrictions. A significant difference in all of the treatment restrictions was found between the patients who died and those who survived. 66 patients (83.5%) that died during their admission had a ‘do not resuscitate’ restriction versus 136 patients (63.8%) who survived (*p*-value 0.020), 66 patients (83.4%) that died had a ‘do not intubate’ restriction versus 129 (60.6%) of the patients who survived (p 0.014) and 63 patients (79.7%) in the mortality group had a ‘no ICU admission’ restriction versus 122 patients (57.3%) in the survival group (*p*-value 0.007).

The CCI scores are comparable between the groups, with a median of 5 in both subgroups, but with a higher minimum in the group of in-hospital mortality, which was also found to be significant (*p*-value 0.040). We made a subdivision between high and low CCI-scores. The results show significantly higher CCI-score (≥ 5) are more often found in the group of in-hospital mortality (69.6% versus 56.8%) and that a lower CCI [1–4] is more often found in the survival group (42.3% versus 29.1%), p-value 0.042.

### Associations with in-hospital mortality

Table 3 shows the association of APOP items and in-hospital mortality. There was an elevated risk of in-hospital mortality when patients scored both high risk of functional and evidence of cognitive impairment (OR 2.24, 95% 1.18–4.25), compared to the low risk reference group. The high risk of functional decline, shows the highest elevation of risk for in-hospital mortality, but the result was not significant. The APOP result of evidence of cognitive impairment did not show a higher risk of in-hospital mortality compared to the reference group.

**Table 3:** Odds Ratio’s for in-hospital mortality

	OR	*95% CI*	*p-value*
APOP			
Low risk on adverse outcomes	Ref.		
High risk functional decline	2.09	0.84–5.22	0.111
Evidence of cognitive impairment	1.57	0.78–3.16	0.210
High risk of functional decline and evidence of impaired cognition	2.24	1.18–4.25	0.014
CFS			
Fit [1–3]	Ref.		
Vulnerable [4]	0.64	0.26–1,55	0.324
Frail [5–9]	1.35	0.75–2.47	0.321
Charlson Comorbidity Index			
CCI 1–4	Ref.		
CCI ≥ 5	1.78	1.02–3.11	0.043
Subgroup ‘No treatment restrictions (*n* = 67)’			
Low risk on adverse outcomes	Ref.		
High risk functional decline	< 0.01	0.0	0.999
Evidence of cognitive impairment	2.73	0.65–11.56	0.172
High risk of functional decline and evidence of impaired cognition	6.83	0.81–58.02	0.078

*APOP* Acute Presenting Older Patient-screening, *CFS* Clinical Frailty Scale

The CFS did not show a significant elevated risk of in-hospital mortality when comparing vulnerable or frail patients to fit patients. The vulnerable category even shows a somewhat protective effect, although these results are not significant (OR 0.64, 95% 0.26–1.55).

The odds of in-hospital mortality were 78% higher in patients with high CCI-scores (5 and higher) compared to the lower CCI-scores. This elevated risk was found to be significant (OR 1.78, 95% CI 1.02–3.11, *p*-value 0.043).

In the subgroup analysis consisting of patients with no treatment restrictions (*n* = 67), 10 patients died during their hospital stay (14.9%). The APOP result ‘evidence of cognitive impairment’ was associated with an elevated risk of in-hospital mortality (OR 2.73, 95% CI 0.65–11.56, *p* 0.172). The combined score consisting of both evidence of impaired cognition and high risk of functional decline increased risk of in-hospital mortality even more (OR 6.83, CI 95% 0.81–58.02, *p* 0.078). However, both are not proven to be significant in this specific group and thus these results should be interpreted with caution. The low odd’s ratio in the high risk of functional decline group is due to the small size of patients who had no treatment restriction. There were only two patients with high risk of functional decline within this group, both survived.

### Predictive values of the frailty instruments and CCI

To compare the predictive value of the APOP-screener to the CFS and CCI, we calculated AUCs for both frailty measurements and the CCI. (Table 4) The AUC of the APOP-screener was 0.59, with 95% CI between 0.52 and 0.66. The CFS showed a comparable AUC of 0.54, 95% CI 0.46–0.62. The AUC of the CCI was calculated as a continuous scale as well as into two categories. Both the continuous and categorical variant are comparable to the frailty instruments, with the continuous CCI showing an AUC of 0.58 (95% CI 0.51–0.65) and the categorical CCI showing an AUC of 0.57 (95% 0.49–0.64).

**Table 4:** Comparison of the APOP-screener with the Clinical Frailty Scale and CCI

Variables	In hospital mortality	
	AUC	95% CI
APOP	0.59	0.52–0.66
CFS 3 categories	0.54	0.46–0.62
CCI (continuous)	0.58	0.51–0.65
Categorical CCI (CCI 1–4 and CCI ≥ 5)	0.57	0.49–0.64

*APOP* Acute presenting Older Patient-screening, *CFS* Clinical Frailty Scale

## Discussion

In this retrospective study, we evaluated the APOP-screener for its ability to predict in-hospital mortality in older ED-patients with COVID-19. We compared this frailty measurement to the worldwide accepted Clinical Frailty Scale and the Charlson Comorbidity Index. The APOP-screener showed a significantly increased risk for in hospital mortality if patients showed a high risk of functional decline and evidence of cognitive impairment (OR 2.24, 95% 1.18–4.25, *p*-value 0.014). In the subgroup analysis of patients without treatment restrictions this effect lost significance. Treatment-restrictions appear to be possible confounders and underestimate the association between frailty and in-hospital mortality. Due to the relatively small sample size, effects could be under or over estimated as indicated by the broad confidence intervals. This could also explain why well researched risk factors as high BMI and chronic kidney disease, are not statistically different in both groups. A larger, most preferably multicentre population is needed to evaluate the true predictive value of the APOP-score. Although an increased risk for in-hospital mortality was found for the most-frail patients as scored by the APOP and for the most comorbid patients as scored by the CCI, these scoring instruments had poor discriminatory value. The CFS did not show significance in predicting in-hospital mortality and had a poor discriminatory value as well. Therefore, treatment decisions based on frailty or comorbidities alone should be made with caution and a broader view/approach is needed.

With the burden that COVID-19 has placed on health care systems around the world, identifying patients from who the burdens of aggressive supportive treatment outweigh the benefits is a worldwide subject of interest. With the variety of clinical presentation of patients infected with COVID-19 and the heterogeneous population it affects, identifying risk factors is difficult and a sufficiently accurate model for predicting poor outcomes in COVID-19 does not yet exist. With advancing age being associated with high mortality rates, most often restrictive recommendations have been directed broadly to people older than 70 years or older, irrespective of risk-factors, health or activity status [8]. However, a recent study showed that for the patients aged 80 years and above further increasing age is not a risk factor for survival, whereas the presence of dementia and indicators for a severe COVID-19 infection (low saturation, high lactate dehydrogenase) are risk factors for death [35].

When evaluating predictive factors for poor outcomes, it is of upmost importance to keep the large heterogeneity of the older population into account. Due to the variety of age and comorbidities but also resilience, susceptibility for disease and vulnerability within this population, a prediction model solely based on one of these factors will lack prognostic value. The concept of frailty attempts to incorporate the existing variabilities in this population. Although many frailty assessment instruments are available, relatively few are validated for use in the acute inpatient setting. In this study we proposed the APOP-screener as a possible predictive factor for poor outcomes in COVID-19 and compared this with the CFS. Based on previous research the APOP-screener might be a promising assessment to identify elderly at highest risk for adverse outcomes during this global pandemic [15, 16]. Its ability to predict 3-month mortality in elderly at the emergency department has already been proven. However, the APOP-screener has not been validated for in-hospital mortality, nor has it been evaluated in COVID-infected patients. The APOP-screener is a standardized instrument of measuring frailty at the ED, by using standardized questions without the need of interpreting the results and has already been implemented for screening frailty at the ED in various hospitals in the Netherlands, where it proved to be feasible in daily clinical practice. This screener is validated for 30 day and 3-month mortality, but has not been validated yet for in-hospital mortality. In this study, the APOP-screener showed a two-fold increase in risk of in hospital mortality if patients showed a high risk of functional decline and evidence of cognitive impairment. However, this effect was lost when evaluated in the subgroup of no treatment restrictions. These outcomes suggest that the APOP might be a promising screening tool, however a larger, most preferably multicentre population is needed to evaluate the true predictive value of the APOP-screener. Limitations of the APOP-screener is the active patient participation in two of the nine needed questions, which could be difficult in severely ill patients, and the fact that it has only been validated in patients of 70 years or older.

The Clinical Frailty Scale is another frailty measurement used in acute care settings worldwide. This instrument does not rely on active patient-participation and the value of predicting mortality has already been established. The CFS is the most studied instrument in COVID-19 patients and has already been implemented in different ethical guidelines during this pandemic [36]. A recent meta-analysis, consisting of two prospective and six retrospective cohorts by Pranata et al., shows that the CFS is significantly associated with an increase in mortality [13]. This relationship could not be found in this study population. An advantage of the CFS is that it can be assessed from a short (hetero) anamnesis, and thus is very useful in hyperacute settings. This instrument has a high reliance on proxies for information and requires an active patient or family/caretaker involvement. Because of its subjective nature, inter-rater variability could exist. In recent published studies by Flaatten et al. and Surkan et al., a fair interrater reliability of the CFS was found if assessed by ICU-personnel and geriatricians [37–39]. However, in times of a pandemic when physicians or nurses with minimal experience in geriatric care are needed to assess these patients and use the CFS, we expect a higher interrater variability due to the subjective nature of this instrument.

When comparing the results of the APOP-screener and the CFS, overlap stands out, especially in the highest and lowest categories of both (Fig. 1). Discrepancies are mostly present in the APOP category ‘evidence of impaired cognition’, whereby nearly a quarter of those patients fall in the ‘fit’ category in the CFS. This could be due to the fact that in milder forms of cognitive impairment, people might not yet experience interruptions in daily life (as measured by the CFS), but might be unable to answer cognitive questions as been asked in the APOP-screener.

With advancing age comes a higher prevalence of chronic conditions, and a widely accepted scoring method for measuring comorbidities is the CCI [9]. In previous studies, the CCI has been evaluated for predicting mortality in COVID-19 patients and proven independently associated with mortality, with a higher score indicating more severe comorbid conditions and a greater mortality rate [8, 10, 11]. In our study population, the odds of in-hospital mortality were significantly higher in patients with high CCI-scores compared to the lower CCI-scores. However, when looking at the predictive value of the CCI in our population, this was moderate and comparable to the APOP screener and the CFS. Like the APOP and CFS, the Charlson Comorbidity Score attempts to incorporate existing variabilities in the older population, but unfortunately it is not enough to predict in-hospital mortality by itself.

Frailty as an approximation of the existing variability of the older population is associated with a higher risk of adverse outcomes as functional impairment, hospitalization and mortality in general. Despite our expectations, both frailty instruments, proved average in predicting survival in older COVID-19 patients. This raises the question if, in accordance with the statement of the Royal Dutch Medical Association (RDMA), frailty measurements should be used in allocating medical care in times of a pandemic [40]. In times of extreme shortage of ICU beds, prioritizing systems need to be constructed to guide clinicians. In this situation, an imbalance exists between individual ethics and societal ethics: ones wish to take every opportunity to survive and the societal need to provide specialized health care for the ones with the best chances [41]. In this light, it is important to provide robust scientific evidence to support assumptions about predictors for survival. Although frailty has already been implemented in different ethical guidelines during this pandemic, the results demonstrated in our study might suggest that further research is needed to demonstrate the predictive value of frailty in COVID-19 infected older patients. Because of the likelihood of the association between frailty and mortality in earlier research, assessments to identify frailty could be valuable.

Until this day, there is no golden standard in predicting adverse outcomes in COVID-19 infected older patients. Future research could focus on combining a frailty instrument, such as the APOP-screener with other predictive factors. This concept is evaluated in a recent study by Hägg et al., which showed that higher age is associated with in-hospital mortality and decreased probability of discharged back to home in geriatric patients, and including frailty and comorbidity assessments to the models improves their predictive accuracy. When combined with objective measurements, for example the recently developed RISE-UP score (AUC 0.83 for 30 day mortality in COVID-19 patients), we hypothesize this might be the multidimensional prediction model that is needed to identify older patients with COVID-19 who are at risk of adverse outcomes [42].

### Limitation

Our study had several limitations. First of all, our data were retrospectively collected using an algorithm in the electronic medical records and patients were not approached for any additional data, which leads to missing data for some baseline characteristics and outcomes. Additionally, by assessing the CFS retrospectively according to the described functional status, living arrangement and need of care, this could lead to information bias. Retrospective scoring of the CFS, solely based on inpatient records, has been studied by several researchers in different (non-COVID-19) populations. In these studies, interrater reliability between face-to-face assessment and inpatient records proved substantial to high, with kappa values ranging between 0.64 and 0.89 [25–29]. This study, in agreement with most research with the CFS, divided in 3 categories in this study, creating groups that are large enough for analysis. Dividing the CFS in categories does not seem to lead to less than substantial inter-rater reliability between face-to-face assessment and inpatient records [25, 27, 29]. In our cohort there were no patients classified with an CFS-score of 9 (terminally ill), which could induce bias. However, it is to be assumed that terminally ill patients won’t be referred to the ED when being diagnosed with a COVID-19 infection, because of the already poor prognosis and adverse outcomes in this group of patients. ​ The study population consists of patients from a single medical centre, located in one of the most severely affected areas in the Netherlands. In contrast to the CFS, the APOP-screener is a measurement instrument that requires active participation from the patient, which results in worse applicability in critically ill patients. With the defined inclusion-criteria for this study, only patients with a complete APOP-score were included which might have caused selection bias, leading to a relatively low mortality rate in our study. Additionally, the APOP was used as a categorical measurement, adopting a cut-off value validated in multiple non-COVID cohorts [23]. A different cut-off value could possibly enhance the predictive value of the APOP-screener in older patients with COVID-19.

The strength of this study is the sample size of almost 300 consecutive patients, and by analysing the screening instruments in the same cohort, there were no differences in population characteristics. However, our study-population is not suitable for validating a new (combined) prediction model due to its single-centred approach.

## Conclusion

Although the elevated risk for in-hospital mortality found for the most-frail patients as scored by the APOP and the most comorbid patients as scored by the CCI, both had poor discriminatory value. The CFS did not show significance in predicting in-hospital mortality. Prediction models can be useful for identifying patients at high risk for poor outcomes and may guide clinical-decision making. However, treatment decisions based on frailty screening alone should be made with caution. Approaching the heterogeneity of the older population by adding frailty as assessed by the APOP-score to existing prediction models may enhance the predictive value of these models.

## Supplementary Information

**Additional file 1 Table S1.** Queries used in CTcue.

## Data Availability

Additional data are available upon reasonable request. Data can be requested by contacting Marleen GAM van der Velde (corresponding author).

## References

[1] Yang J, Tian C, Chen Y, Zhu C, Chi H, Li J (2020). Obesity aggravates COVID-19: an updated systematic review and meta-analysis. J Med Virol..

[2] Booth A, Reed AB, Ponzo S, Yassaee A, Aral M, Plans D (2021). Population risk factors for severe disease and mortality in COVID-19: a global systematic review and meta-analysis. PLoS One.

[3] Patel U, Malik P, Mehta D, Shah D, Kelkar R, Pinto C (2020). Early epidemiological indicators, outcomes, and interventions of COVID-19 pandemic: a systematic review. J Glob Health.

[4] Zhou Y, Yang Q, Chi J, Dong B, Lv W, Shen L (2020). Comorbidities and the risk of severe or fatal outcomes associated with coronavirus disease 2019: a systematic review and meta-analysis. Int J Infect Dis.

[5] Izcovich A, Ragusa MA, Tortosa F, Lavena Marzio MA, Agnoletti C, Bengolea A (2020). Prognostic factors for severity and mortality in patients infected with COVID-19: a systematic review. PLoS One.

[6] Qiu P, Zhou Y, Wang F, Wang H, Zhang M, Pan X (2020). Clinical characteristics, laboratory outcome characteristics, comorbidities, and complications of related COVID-19 deceased: a systematic review and meta-analysis. Aging Clin Exp Res.

[7] Wynants L, Van Calster B, Collins GS, Riley RD, Heinze G, Schuit E (2020). Prediction models for diagnosis and prognosis of covid-19: systematic review and critical appraisal. BMJ.

[8] Hägg S, Jylhävä J, Wang Y, Xu H, Metzner C, Annetorp M (2020). Age, frailty, and comorbidity as prognostic factors for short-term outcomes in patients with coronavirus disease 2019 in geriatric care. J Am Med Dir Assoc.

[9] Charlson ME, Pompei P, Ales KL, MacKenzie CR (1987). A new method of classifying prognostic comorbidity in longitudinal studies: development and validation. J Chronic Dis.

[10] Imam Z, Odish F, Gill I, O'Connor D, Armstrong J, Vanood A (2020). Older age and comorbidity are independent mortality predictors in a large cohort of 1305 COVID-19 patients in Michigan, United States. J Intern Med.

[11] Tuty Kuswardhani RA, Henrina J, Pranata R, Anthonius Lim M, Lawrensia S, Suastika K (2020). Charlson comorbidity index and a composite of poor outcomes in COVID-19 patients: a systematic review and meta-analysis. Diabetes Metab Syndrome.

[12] Church S, Rogers E, Rockwood K, Theou O (2020). A scoping review of the clinical frailty scale. BMC Geriatr.

[13] Pranata R, Henrina J, Lim MA, Lawrensia S, Yonas E, Vania R (2021). Clinical frailty scale and mortality in COVID-19: a systematic review and dose-response meta-analysis. Arch Gerontol Geriatr.

[14] Blomaard LC, van der Linden CMJ, van der Bol JM, Jansen SWM, Polinder-Bos HA, Willems HC (2021). Frailty is associated with in-hospital mortality in older hospitalised COVID-19 patients in the Netherlands: the COVID-OLD study. Age Ageing..

[15] Sablerolles RSG, Lafeber M, van Kempen JAL, van de Loo BPA, Boersma E, Rietdijk WJR (2021). Association between clinical frailty scale score and hospital mortality in adult patients with COVID-19 (COMET): an international, multicentre, retrospective, observational cohort study. Lancet Healthy Longev.

[16] O'Caoimh R, Costello M, Small C, Spooner L, Flannery A, O'Reilly L (2019). Comparison of Frailty Screening Instruments in the Emergency Department. Int J Environ Res Public Health.

[17] Rockwood K, Song X, MacKnight C, Bergman H, Hogan DB, McDowell I (2005). A global clinical measure of fitness and frailty in elderly people. CMAJ.

[18] Anand A, Cudmore S, Robertson S, Stephen J, Haga K, Weir CJ (2020). Frailty assessment and risk prediction by GRACE score in older patients with acute myocardial infarction. BMC Geriatr.

[19] Muessig JM, Nia AM, Masyuk M, Lauten A, Sacher AL, Brenner T (2018). Clinical frailty scale (CFS) reliably stratifies octogenarians in German ICUs: a multicentre prospective cohort study. BMC Geriatr.

[20] Ritt M, Ritt JI, Sieber CC, Gaßmann K-G (2017). Comparing the predictive accuracy of frailty, comorbidity, and disability for mortality: a 1-year follow-up in patients hospitalized in geriatric wards. Clin Interv Aging.

[21] de Gelder J, Lucke JA, de Groot B, Fogteloo A, Anten S, Mesri K (2016). Predicting adverse health outcomes in older emergency department patients: the APOP study. Neth J Med.

[22] CTcue. <https://ctcue.com/>. Accessed on 7-12-2021.

[23] de Gelder J, Lucke JA, Blomaard L, Booijen A, Fogteloo A, Anten S (2018). Optimization of the APOP screener to predict functional decline or mortality in older emergency department patients: cross-validation in four prospective cohorts. Exp Gerontol.

[24] Blomaard L, Lucke J, de Gelder J, Anten S, Alsma J, Schuit S (2020). The APOP screener and clinical outcomes in older hospitalised internal medicine patients. Neth J Med.

[25] Marincowitz C, Turner V, Allgar V, Bellwood J, Wheeler A, Hale M (2020). Can patient frailty be estimated from inpatient records? A prospective cohort study. Advanc Geriatr Med Res.

[26] Davies J, Whitlock J, Gutmanis I, Kane SL (2018). Inter-rater reliability of the retrospectively assigned clinical frailty scale score in a geriatric outreach population. Can Geriatr J.

[27] Stille K, Temmel N, Hepp J, Herget-Rosenthal S (2020). Validation of the clinical frailty scale for retrospective use in acute care. Eur Geriatr Med.

[28] Fornaess KM, Nome PL, Aakre EK, Hegvik TA, Jammer I (2022). Clinical frailty scale: inter-rater reliability of retrospective scoring in emergency abdominal surgery. Acta Anaesthesiol Scand.

[29] Darvall JN, Boonstra T, Norman J, Murphy D, Bailey M, Iwashyna TJ (2019). Retrospective frailty determination in critical illness from a review of the intensive care unit clinical record. Anaesth Intensive Care.

[30] O’Caoimh R, Gao Y, Svendrovski A, Healy E, O’Connell E, O’Keeffe G (2014). Screening for markers of frailty and perceived risk of adverse outcomes using the risk instrument for screening in the community (RISC). BMC Geriatr.

[31] Andersen FH, Ariansen Haaland Ø, Klepstad P, Flaatten H (2021). Frailty and survival in elderly intensive care patients in Norway. Acta Anaesthesiol Scand.

[32] Christensen DM, Strange JE, Gislason G, Torp-Pedersen C, Gerds T, Fosbøl E (2020). Charlson comorbidity index score and risk of severe outcome and death in Danish COVID-19 patients. J Gen Intern Med.

[33] Testa G, Cacciatore F, Galizia G, Della-Morte D, Mazzella F, Russo S (2009). Charlson comorbidity index does not predict long-term mortality in elderly subjects with chronic heart failure. Age Ageing.

[34] Iaccarino G, Grassi G, Borghi C, Ferri C, Salvetti M, Volpe M (2020). Age and Multimorbidity Predict Death Among COVID-19 Patients: Results of the SARS-RAS Study of the Italian Society of Hypertension. Hypertension (Dallas, Tex: 1979).

[35] Covino M, De Matteis G, Santoro M, Sabia L, Simeoni B, Candelli M (2020). Clinical characteristics and prognostic factors in COVID-19 patients aged ≥80 years. Geriatr Gerontol Int.

[36] Hewitt J, Carter B, Vilches-Moraga A, Quinn TJ, Braude P, Verduri A (2020). The effect of frailty on survival in patients with COVID-19 (COPE): a multicentre, European, observational cohort study. Lancet Public Health.

[37] Surkan M, Rajabali N, Bagshaw SM, Wang X, Rolfson D (2020). Interrater reliability of the clinical frailty scale by geriatrician and Intensivist in patients admitted to the intensive care unit. Can Geriatr J..

[38] Pugh RJ, Ellison A, Pye K, Subbe CP, Thorpe CM, Lone NI (2018). Feasibility and reliability of frailty assessment in the critically ill: a systematic review. Crit Care.

[39] Flaatten H, Guidet B, Andersen FH, Artigas A, Cecconi M, Boumendil A (2021). Reliability of the clinical frailty scale in very elderly ICU patients: a prospective European study. Ann Intensive Care.

[40] KNMG & Federatie Medisch Specialisten, Draaiboek ‘Triage op basis van niet-medische overwegingen voor IC-opname ten tijde van fase 3 in de COVID-19 pandemie. [Internet]. 2020 [cited 24-03-2021]. Available from: https://www.rijksoverheid.nl/documenten/publicaties/2020/06/16/draaiboek-triage-op-basis-van-niet-medische-overwegingen-voor-ic-opname-ten-tijde-van-fase-3-in-de-covid-19-pandemie.

[41] Robert R, Kentish-Barnes N, Boyer A, Laurent A, Azoulay E, Reignier J (2020). Ethical dilemmas due to the Covid-19 pandemic. Ann Intensive Care.

[42] van Dam PM, Zelis N, Stassen P, van Twist DJL, De Leeuw PW, van Kuijk S (2021). Validating the RISE UP score for predicting prognosis in patients with COVID-19 in the emergency department: a retrospective study. BMJ Open.

