# Supplementary material for: Performance of the APOP-screener for predicting in-hospital mortality in older COVID-19 patients: a retrospective study
Source: BMC Geriatr. 2022 Jul 15;22:584. doi: 10.1186/s12877-022-03274-2 (PMC9284964; doi:10.1186/s12877-022-03274-2)
Supplement: Supplementary file 1 — Additional file 1 Table S1. Queries used in CTcue. [file 12877_2022_3274_MOESM1_ESM.pdf]

Supplementary appendix

Table S1: Queries used in CTcue

**Characteristics in CTcue**	**Where**	**Specified**	**Specified (translated)**
Gender		Male<br>Female	
Age		At the time of data collection<br>At the time of the ED-visit	
Birthyear			
Mortality		In-hospital mortality<br>Overall mortality	
Date of positive COVID-test			
Date of ED-visit in relation to the positive COVID-test			
Date of APOP-screening in relation to the COVID-test and ED-visit			
Length + date			
Weight + date			
BMI + date			
Smoking		Never<br>Ever<br>Yes	
Transport to ED		Ambulance<br>Own transport	
Living arrangement		"Sociale status"	Social anamnesis
Home care		"Thuiszorg/HDL/ADL"	Home care, activities of daily living, instrumental activities of daily living
COVID samples	Microbiology	Naso/oropharynx<br>Sputum<br>BAL<br>Feces	
Influenza samples	Microbiology		
RS samples	Microbiology		
APOP screening + outcome			
CFS		"ADL/BDL/mantelzorg/AR"	Home care, care takers, activities of daily living,

			instrumental activities of daily living, functional status, use of tools (walker etc.)
Voorgeschiedenis	Patients history	According to a patients history scored for CCI	
Symptoms	Anamnesis	Cough, sputum, fever, malaise	
Days of symptoms	Anamnesis	According to anamnesis	
Vital parameters		HR, RR (systolic and diastolic), respiratory rate, saturation, O2-supply	
Bloodgas		pH, pCO2, pO2, bicarbonate, base excess, sO2	
Blood tests		CRP, trombocytes, leukcocytes, lymfocytes abs, PT, APTT, D-dimeer, procalcitonine, potassium, creatinine, eGFR, ALAT, ASAT, lactate, CK, LD	
X-thorax	Radiology	"Consolidations, infiltration"	
Treatment restrictions		NR<br>NB<br>IC-<br>And combinations of these options	Do not resuscitate<br>Do not ventilate<br>No ICU admission
Location after ED-visit		Home<br>Admission	
Location of admission		COVID-cohort<br>Internal medicine<br>Pulmonary medicine	
Duration of admission in days			
ICU-admission		Yes<br>No	
Location after admission		Home<br>ELV<br>Revalidatie (GRZ)<br>Hospice	Home<br>Rehabilitation<br>Care/nursing home<br>End of life care
Revisits (+ reason) after ED-visit related to COVID-infection			
